# Thermal Stability, Fire Performance, and Mechanical Properties of Natural Fibre Fabric-Reinforced Polymer Composites with Different Fire Retardants

**DOI:** 10.3390/polym11040699

**Published:** 2019-04-16

**Authors:** Erik Valentine Bachtiar, Katarzyna Kurkowiak, Libo Yan, Bohumil Kasal, Torsten Kolb

**Affiliations:** 1 Centre for Light and Environmentally-Friendly Structures, Fraunhofer Wilhelm-Klauditz-Institut WKI, Bienroder Weg 54E, Braunschweig 38108, Germany; erik.bachtiar@wki.fraunhofer.de (E.V.B.); bohumil.kasal@wki.fraunhofer.de (B.K.); torsten.kolb@wki.fraunhofer.de (T.K.); 2 Department of Organic and Wood-Based Construction Materials, Technical University of Braunschweig, Hopfengarten 20, 38102 Braunschweig, Germany; katarzyna.kurkowiak@o2.pl

**Keywords:** aluminum hydroxide, ammonium polyphosphate, flame retardants, limited oxygen index, natural flax fiber reinforcement, mechanical tensile test, polymer composites, thermogravimetric analysis, Underwriters Laboratories (UL)-94 test

## Abstract

In this study, ammonium polyphosphate (APP) and aluminum hydroxide (ALH) with different mass contents were used as fire retardants (FRs) on plant-based natural flax fabric-reinforced polymer (FFRP) composites. Thermogravimetric analysis (TGA), limited oxygen index (LOI), and the Underwriters Laboratories (UL)-94 horizontal and vertical tests were carried out for evaluating the effectiveness of these FR treatments. Flat-coupon tensile test was performed to evaluate the effects of FR treatment on the mechanical properties of the FFRP composites. For both fire retardants, the results showed that the temperature of the thermal decomposition and the LOI values of the composites increased as the FR content increases. Under the UL-94 vertical test, the FFRP composites with 20% and 30% APP (i.e., by mass content of epoxy polymer matrix) were self-extinguished within 30 and 10 s following the removal of the flame without any burning drops, respectively. However, the mechanical tensile tests showed that the APP treated FFRP composites reduced their elastic modulus and strength up to 24% and 18%, respectively. Scanning electronic microscopic (SEM) for morphology examination showed an effective coating of the flax fibres with the FRs, which improved the flame retardancy of the treated composites.

## 1. Introduction

Plant-based natural fibre-reinforced polymer (NFRP) composites have attracted increased attention as a replacement of synthetic fibre-reinforced polymer composites in various engineering applications. This is because of the biodegradability, low price, energy efficiency, low carbon footprint, and good specific tensile properties of these composites. Among dozens of plant-based natural fibres, flax fibres show promising potential because of their favourable combination of strength and stiffness, low cost, and relatively high annual production of the fibres. Specific mechanical properties (e.g., specific tensile strength and specific tensile modulus of flax fibres) are comparable to those of synthetic E-glass fibre [1]. This comparison is, however, somewhat misleading, since the natural fibres are not endless (compared to glass or carbon fibers) but are used in the form of yarns, which will generally have lower mechanical properties compared to the ones of individual fibres. Hemp and flax are still widely cultivated in France, the Netherlands, and some eastern European countries. It is estimated that the market of NFRP composites will reach up to 5.2 billion euros in 2019, and this value will steadily increase in the following years [2].

NFRP composites have been researched for various applications such as civil engineering, automotive engineering, and aircraft and aviation engineering [3,4,5]. In civil engineering, NFRP composites were proven to be effective as external confinement in concrete columns [6,7,8,9] and external flexural strengthening reinforcement for concrete beams [10,11]. In the automotive industry, the NFRP composites were used as interior and exterior components for vehicles, such as door panels, side panels, headliners, dashboard parts, back side of seats, truck liners, and many others [4,12]. NFRP composites were also investigated as energy absorbers for the crashworthiness design of vehicles [13,14]. In aircraft and aviation engineering, flax woven-reinforced phenolic composite panels have been investigated for cabin interiors [15], and aircraft wing-box structures have been designed from natural ramie fibre-reinforced epoxy composite [16].

Although there are some promising examples of the applications of NFRP composites for different engineering purposes, some critical drawbacks of using natural fibres as reinforcement of polymer composites still exist. The plant-based natural fibres are hydrophilic, which is incompatible with hydrophobic polymer matrices. This leads to a reduction of their interfacial bonds and to the poor mechanical properties of the resulting fibre-reinforced polymer composite. The low processing temperature and poor thermal stability of plant-based natural fibres are also suspected to negatively influence their long-term durability [1,17]. To improve fibre-polymer interfacial bonding and their composite thermal stabilities, different treatments of the fibre surface or the polymer matrix have been proposed. Adhesion can be improved by physical modifications of the fibres (e.g., stretching, calendaring, electric discharge, and mercerization) as well as by various chemical modifications (e.g., chemical coupling, graft copolymerization, impregnation of fibres, or treatment with isocyanates) [18]. Thermal stability can be improved with chemical treatment (e.g., copolymerization and grafting) [19].

Another critical issue is the fire performance of NFRP composites. Plant-based natural fibres are highly flammable. Therefore, their fibre-reinforced polymer composites need to be protected against fire if used in applications such as in automotive, civil, or aircraft industries. It is believed that the addition of fire retardants (FRs) has the potential to expand the use of NFRP composites [19,20,21]. The use of FRs for NFRP composites may inhibit or even suppress the combustion processes, for example during heating, decomposition, ignition, and flame spread [21]. Studies have shown that it is possible to enhance the fire resistance of NFRP composites by the incorporation of fire-retardant additives such as halogen, nitrogen and phosphorus-based compounds [22,23,24].

Halogenated additives can prevent flame spread, but they also generate dense smoke and corrosive combustion by-products, which negatively impacts the environment and fire safety [22]. Depending on the polymer, additives, and fire conditions, gases such as hydrogen chloride (HCl), hydrogen fluoride (HF), hydrogen cyanide (HCN), and carbon monoxide (CO) may be produced in varying quantities from a burning composite. These compounds are considered toxic even at relatively low concentrations [23,24]. Phosphorus-based fire retardants are considered safer alternatives. Loredo et al. [22] treated flax fibre-reinforced polyester composites with Exolit 470 [25], which is a mixture of ammonium polyphosphate and other intumescent compounds. The composites exhibited a high fire resistance with a significant reduction in the peak heat release rate, total heat release rate, and maximum average of heat emission values. In the same study, the combination of ammonium polyphosphate and alumina trihydrate was also tested. The results showed their potential as fire retardants. 

The complex nature of the flammability of modified composites was examined by Szolnoki et al., Lim et al., and Crossley et al. [26,27,28]. Szolnoki et al. [26] treated natural hemp fabric-reinforced epoxy resin composites by the immersion of preheated fabric into a cold phosphoric acid solution and replacing the standard curing agent of the epoxy with an aminosilane-type coupling agent. The fire resistance of the treated hemp fabric/epoxy matrix composite increased to a V-0 rating [29] (self-extinguished within 10 s after the removal of the flame, without burning drops). However, the tensile performance was much poorer, when only one of the components (either matrix or fibres) was treated with the FR. This was suspected to be to the result of a better adhesion between the modified fibres and the matrix as well as improved wettability of the fibres. The impact of an ammonium polyphosphate (APP)-based compound, Budit 3167 [30], on kenaf fibre-reinforced polypropylene composites was investigated by Lim et al. [27]. The improvement of fire resistance with a UL-94 V-0 rating [29], lower peak heat release rate, higher time to ignition, and relatively small smoke production rates were observed. Additionally, better tensile and flexural moduli were obtained, since the flame retardant also acted as a particle reinforcement. However, the quality of fibre/matrix interfacial bonds was reduced, which led to a decline in flexural strength in comparison to the referenced composites. Crossley et al. [28] manufactured renewable furan resin composites reinforced by flax fibres and compared them with the flax fibre-reinforced polyester, epoxy, and phenol composites. The tensile and flexural strengths turned out to be 38–77% and 11–95% lower in comparison to composites with other mentioned polymer matrixes. Additionally, large voids were found at the fibre/matrix interface. Under horizontal burn testing, the flax/furan composite self-extinguished after 10 s with minimal smoke. However, the composite failed the vertical burn test. The phenol resin, on the other hand, improved the flax/phenol laminate flammability effectively, and the material passed the horizontal and vertical burn tests [28].

As a further enhancement, the current study investigates the fire performance of natural flax fibre fabric-reinforced polymer (FFRP) composites with and without fire retardants. Ammonium polyphosphate (APP) and aluminium hydroxide (ALH) were used as the fire retardants. To determine their thermal stability and fire performance, the composites were evaluated by thermogravimetric analysis (TGA), limiting oxygen index (LOI), and Underwriters Laboratories (UL)-94 vertical and horizontal tests. The effects of FRs with different mass contents on the mechanical properties of the natural flax FRP composites were also evaluated under flat-coupon tensile tests. A scanning electron microscopic (SEM) was used to investigate the morphology of the composites. 

## 2. Materials and Methods

### 2.1. Materials

**Figure 1:**
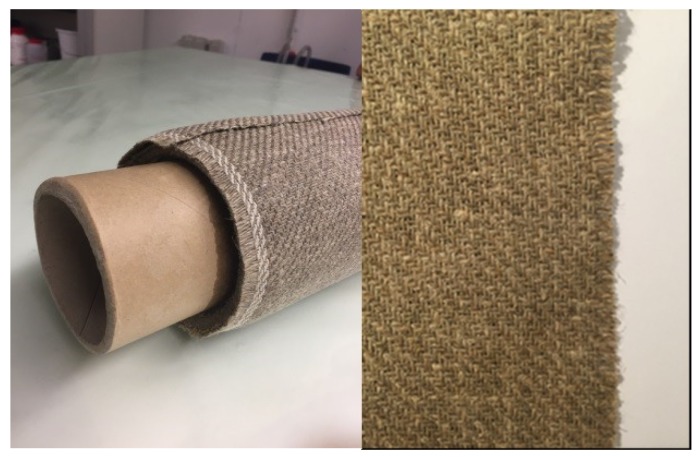
Flax fabric (area density of 550 g m^−2^, 7 yarn threads per cm in fabric warp and weft direction).

A commercial bidirectional flax fabric was obtained from Libeco, Meulebeke, Belgium (with an areal density of 550 g m^−2^) (Figure 1). The average tensile strength and modulus of a single-strand flax yarn were determined to be 153.8 ± 17.5 MPa and 16.4 ± 1.2 GPa, respectively. The polymer matrix was epoxy resin (Prime 20LV resin, Gurit) and the fast hardener (Prime 20 hardener, Gurit). Two fire retardants used for this work were ammonium polyphosphate Exolit^®^ AP 462 produced by Clariant, Muttenz, Switzerland [31] (chemical formula: [NH_4_PO_3_]*n*, *n* > 1000) and aluminium hydroxide produced by Merck Millipore, Darmstadt, Germany [32](chemical formula: Al(OH)_3_·*x*H_2_O).

### 2.2. Sample Preparation

The composite specimens with and without FRs of different type and mass contents of the fire retardant, as listed in Table 1, were produced. The epoxy and hardener were mixed (100:26 by mass ratio) at room temperature with light stirring for 5 min until a homogenous mixture was obtained. For the samples containing a fire retardant (10%, 20%, and 30% for APP or 20%, 30%, and 40% for ALH by mass content of the epoxy), the components of matrix were homogenized in a Dispermat CN20-F2 mixing device [33] with a rotor speed of 300 rpm for 5 min. The flax fibre-reinforced polymer composites were manufactured using a hand lay-up method. The production steps consisted of placing a layer of flax fabric followed by a coating of epoxy mixture (1.26 kg m^−2^) applied using a brush. This step was repeated until the designated layers of the flax fabric were reached. This process allowed a penetration of the epoxy matrix into the flax fabrics. Then, the FFRP composites were cured in room temperature for 24 h. Afterwards, all specimens were stored in a climatic chamber with a relative humidity of 65% ± 5% and a temperature of 20 ± 2 °C for at least 4 d. 

### 2.3. Experiments

#### 2.3.1. TGA (Thermogravimetric Analysis)

Thermal stability of the FFRP composites with and without FR treatment was investigated by means of thermogravimetric analysis using the Thermal Analyzer TGA/DSC 1 STARe System [34]. Each specimen type was tested in both nitrogen and air atmospheres. The weight of the tested specimens were in the range of 10–14 mg, except for 4L-FFRP, which was approximately 35 mg. Each specimen was placed into aluminum oxide crucibles. The chamber containing the test specimens was purified under nitrogen flow (35 mL·min^−1^) for 5 min at 25 °C. Then, the specimen was heated from 25 to 1000 °C, with a heating rate of 10 °C per minute under a constant nitrogen flow of 75 mL·min^−1^ or a constant air flow of 50 mL·min^−1^. The tests were conducted one after another until each type of specimen in both nitrogen and air atmosphere conditions was tested.

#### 2.3.2. LOI (Limiting Oxygen Index)

The fire resistance of the composites was characterized by limited oxygen index tests according to DIN EN ISO 4589-2 [35]. The tests were carried out using an oxygen index instrument LOI Analyzer [36]. The dimensions of the tested composites were 127 mm × 12.7 mm × the thickness of the composite laminates (Table 1). Before testing, the specimens were cured in an environmental chamber with a temperature of 20 ± 2 °C and a relative humidity of 65% ± 5% for 4 d. The minimum oxygen value required to sustain burning was determined by testing a series of specimens. For each specimen type, 2–6 specimens were tested to determine the LOI value.

#### 2.3.3. Vertical and Horizontal Fire Test

Standard UL-94 Vertical (two times flame applications) and UL-94 HB Horizontal flammability tests were performed according to DIN EN 60695-11-10 [29] with the specimen dimensions of 127 mm × 12.7 mm × the thickness of the composite laminates (Table 1). For each type of FR treated FFRP composites, five and three specimens were tested for UL-94 V and for UL-94 HB, respectively. The UL-94 classification was used to determine ignitability and flame spreading rates. The results were graded based on the rating presented in Table 2 and Table 3.

#### 2.3.4. Tensile Test

The tensile tests were carried out based on ASTM D3039 [37] using a universal servo-mechanical testing machine [38]. The composite specimens with dimensions of 250 mm × 25 mm × the thickness of the composite laminates (Table 1) were prepared and tested with a maximum load cell of 100 kN. The test was started by placing the specimen vertically on the testing machine. A load with a displacement rate of 2.5 mm min^−1^ was gradually applied on the specimen until failure. The obtained results were the ultimate strength, the maximum strain, and the elastic modulus.

#### 2.3.5. SEM (Scanning Electron Microscope)

The morphology of flax/epoxy composites with and without APP and ALH fire retardant was examined using a scanning electron microscope (SEM) [39]. Prior to the SEM investigation, each specimen was sputtered with gold using the Bal-Tec SCD-050 sputter coater [40] at room temperature with an acceleration voltage of 420 V for 40 s.

### 2.4. Statistical Analysis

The experimental data obtained in this study were compared via the t-test with a significance level of 0.05. This statistical analysis provided a reliable comparison regarding the differences between these data. Prior to the t-test analysis, other tests were conducted on the normal distribution of the data via Kolmogorov–Smirnov’s test [41] and on the variance equality of each data pair via Levene’s test [42].

## 3. Results and Discussion

### 3.1. TGA (Thermogravimetric Analysis)

**Figure 2:**
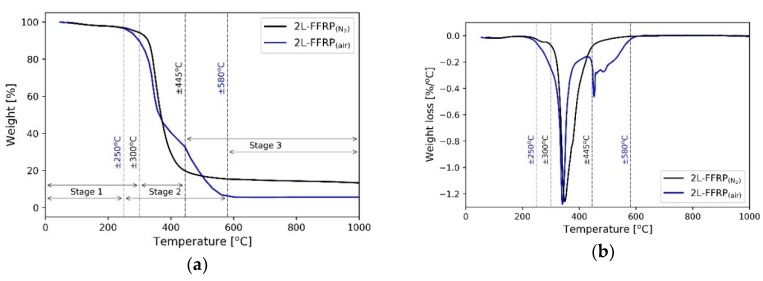
The thermal stability of 2L-FFRP composite tested in air and nitrogen atmosphere conditions: (**a**) thermogravimetric analysis (TGA) curves, (**b**) derivative thermogravimetric (DTG) curves.

**Figure 3:**
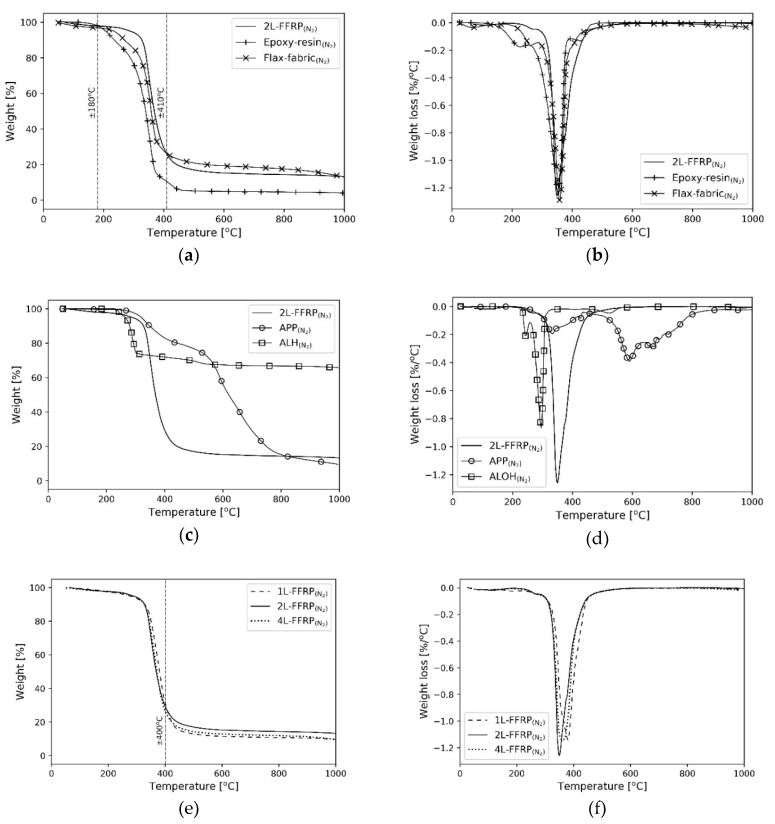
TGA and DTG curves in nitrogen atmosphere of FFRP composite with: (**a**,**b**) flax and epoxy components; (**c**,**d**) fire-retardant compounds; and (**e**,**f**) different number of layers.

**Figure 4:**
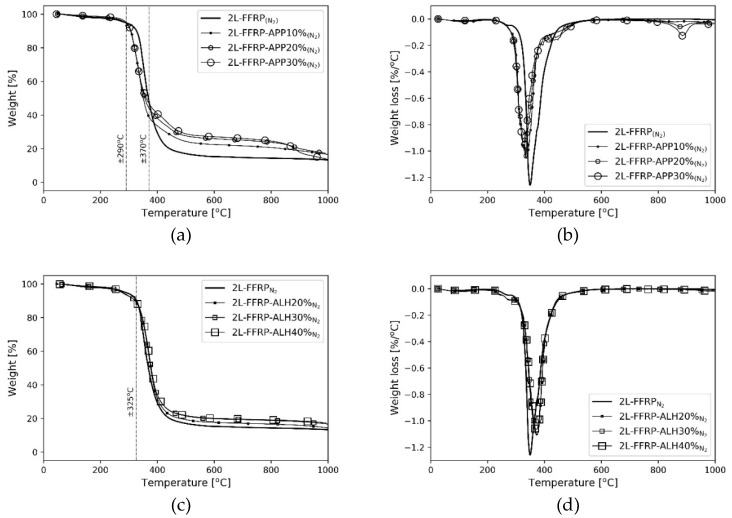

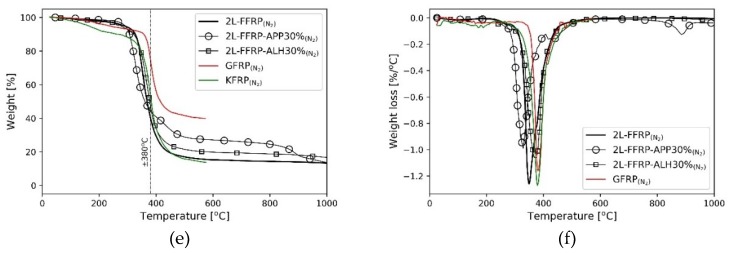
TGA and DTG curves of fire retardant FFRP composites: (**a**,**b**) ammonium polyphosphate (APP); (**c**,**d**) aluminium hydroxide (ALH); and (**e**,**f**) comparison to kenaf and glass fibre-reinforced polymer [47].

The thermal stability and degradation of the selected FFRP composites were studied using TGA in air and nitrogen atmosphere. The results of the TGA and the derivative thermogravimetric (DTG) curves are presented in Figure 2, Figure 3, and Figure 4. In general, thermal degradation of the FFRP composites consisted of three degradation stages (Figure 2a). For the air atmosphere, the first degradation stage was observed when the temperature was lower than 250 °C. In this condition, the weight loss of the specimens mainly resulted from the loss of moisture inside the specimens, which were in the forms of bound water, free water, and water vapour. The second stage, which occurred at temperatures between 250–580 °C, was the stage with the largest weight loss. It was assigned to the degradation of the microstructural component of the material. For a cellulosic material such as flax fibre, the degradation of hemicellulose and cellulose occurred between 200–350 °C, while lignin degraded between 200–500 °C [43]. The third stage, which was at temperatures above 580 °C, was subjected to the degradation of the remaining components. Therefore, very minor weight loss could be observed at this final stage.

Figure 2 also shows the different behaviour of the material in both atmosphere conditions. The test in an air atmosphere (blue line) simulated the degradation behaviour of the material during the application. The material, therefore, was oxidised as it degraded. On the other hand, pure material thermal degradation could be observed for the test in a nitrogen atmosphere (black line). The inert condition of the nitrogen gas protected the material from any other reaction such as oxidation during the degradation process. Several differences were observed based on these results when comparing both atmospheres. In air atmosphere, stage 2 degradation of the composite started at a lower temperature and over a longer temperature range (the temperature range of ±250 to ±580 °C) in comparison to the specimen tested in nitrogen atmosphere (the temperature range of ±320 to ±445 °C). The results can be also observed from the DTG curves presented in Figure 2b. Furthermore, less residue materials were also observed from the specimen tested in air atmosphere because of the oxidation of the char residue.

Figure 3 shows the TGA and DTG results of the FFRP composite, its components, and fire retardant compounds in a nitrogen atmosphere. Figure 3a,b show that the composite had better thermal stability within the range of 180–410 °C compared to their components, flax fabric, and epoxy, separately. This finding suggested a synergistic behaviour between flax and epoxy resin in the composite to obtain a higher resistance in this temperature range. However, the reason remains unknown. The TGA and DTG curves of APP and ALH compounds showed better thermal stability compared to the untreated composite (Figure 3c,d), which confirmed the potential of these materials as fire retardants.

The number of layers of the flax fibres affected the material degradation of the composites (Figure 3e,f). This behaviour was expected because of the dimension of the tested specimens. The specimen with a higher number of layers had to be tested in a higher volume, which led to a lower surface per volume ratio, thus a better thermal stability. However, this reason cannot fully explain the current findings. Below the temperature of 400 °C, the TGA curves of the untreated composites containing one, two, or four layers of flax fibres (2L-FFRP or 4L-FFRP) were almost overlapping each other, which suggested their relatively similar thermal stabilities. At a higher temperature, the composite with two layers of fibre (2L-FFRP) showed better thermal stability. 2L-FFRP could delay material degradation during heating above 400 °C. In addition to the different dimensions of the specimen, slight variations in the absorptivity, specific heat, and thermal conductivity may also contribute to differences of the TGA curves of the tested specimens.

The TGA curves of FFRP composite treated with either APP or ALH fire retardants shifted to a higher temperature in comparison to the untreated ones. The material residue of the treated specimens also increased. These findings suggested an increase of the thermal stability of the specimens (Figure 4). Similar behaviour was also observed in previous studies of these fire retardant materials [44,45]. The influence of the content of fire retardants on the thermal stability of the specimens was also investigated. In nitrogen atmosphere, the increasing content of APP started to influence the degradation process above 370 °C (Figure 4a,b) with a higher content of APP showing better thermal performance. Therefore, out of the three tested concentrations, the composites with 30% APP by mass of epoxy showed the best overall thermal stabilities. The composite containing ALH fire retardant showed improvement in thermal stability for temperatures above 325 °C (Figure 4b,c). Compared to the APP compounds, the higher content of ALH did not always show a better fire performance. The composites with 30% and 40% ALH showed relatively similar degradation rates. During heating, ALH released water vapour. Above 30% ALH, it was suspected that the reaction of water vapours in preventing the degradation of the composite reached a saturation point. Thus, any additional content of the ALH (above 30%) in the material may not have increased its thermal stability.

The composites containing APP and ALH compounds showed relatively similar thermal degradation behaviours up to the temperature of 380 °C (Figure 4e,f). Above this temperature, a different degradation behaviour between ALH and APP compounds was observed. The reason was because of their different mechanisms under high temperatures. The APP material is a chemical fire retardant, which produces protective phosphorus char during heating. This layer of char is much harder to burn, thus, it prevents further combustion. On the other hand, ALH material is a physical fire retardant, which produces aluminum oxide and releases water vapour that increases the heat capacity of the material [46]. The results were also compared to kenaf and glass fibre-reinforced epoxy polymer (KFRP and GFRP) tested in nitrogen atmosphere [47]. The thermal stability of treated FFRP was better than KFRP, and it approached the thermal performance of GFRP.

### 3.2. LOI (Limited Oxygen Index)

In this test, the solid wood specimens of pine wood (*Pinus sylvestris* L.) and beech wood (*Fagus sylvatica* L.), representing soft- and hardwood, were tested as the reference. The results are presented in Table 4. The results show that the LOI of the untreated composites displayed relatively similar values with no significant difference in comparison to both pine and beech wood. The different number of layers of the flax fabrics only slightly affected the LOI value. Among the tested specimens without flame retardant, a higher LOI value of 23.3% (±7.3%) was achieved for the composite with four layers of flax fabric, while an LOI value of 21.3% (±2.7%) was obtained from the one with two layers of fabric. 

The results also showed that the addition of APP and ALH fire retardants to the composites improved their overall fire performances. High LOI values with significant differences to the untreated specimens were obtained from the composite with APP treatment. LOI values of 30.3% (±6.9%) and 25.5% (±12.1%) were achieved by the composite containing 30% and 20% APP, respectively. For comparison, the LOI values of 22%, 21%, and 25% were observed for the untreated hemp, glass, and carbon fabric epoxy composites, respectively, by Marosi et al. [48] and Mizumachi et al. [49]. On the other hand, a similar fire resistance performance could not be achieved by the composite with the ALH treatment. The composite with 40% ALH only reached an LOI value of 24.5% (±2.9%). Although the value was significantly different from the value of 21.3% (±2.7%) achieved by the untreated specimens, the improvement was still rather low. This was because ALH is a physical fire retardant that produces water vapour to increase its fire resistance. In the previous findings by Yang et al. [48], an aluminum hypophosphite compound was used to increase fire resistance of a glass fibre-reinforced polybutylene terephthalate polymer. It was observed that the LOI value increased from 22% to 29%, when 20% of the compound was incorporated in the polymer matrix. The additional phosphate in the fire retardant led to a chemical reaction during heating, thus resulting in the higher LOI values.

In comparison to the solid wood, the LOI of FFRP with 10% APP and 20% ALH showed relatively similar values as the pine wood. The relation of these composites in terms of fire performance to the solid pine wood as a construction material had been also proved by Yan et al. [51]. On the other hand, beech wood showed a slightly higher LOI value of 22.7% (6.7%) compared to the pine wood with an LOI value of 22.0% (4.5%), which suggested a better fire resistance. This was primarily because of the higher density of the wood. Beech wood has an average density of 560 kg·m^−3^, while pine has an average density of only 440 kg·m^−3^ [52]. 

### 3.3. Vertical and Horizontal Fire Test

Sample ignition resistance was investigated under a UL-94 vertical burning test. The rating of each specimen based on Table 2 was assigned after measuring the flame spreading rates and burning times. The test results are presented in Table 5. During the burning test, after-glowing was not observed for any sample. 2L-FFRP-APP30%, assigned a V-0 rating, was the least flammable composite. Similarly, a V-0 rating was also observed on kenaf fiber-reinforced polymer with 30% APP, as investigated by Lim et al. [27]. Even though no UL rating was observed for APP 10% and 20%, the presence of APP significantly decreased the combustion rate of the specimen. Moreover, no specimens treated with ALH reached any rating grade.

The flax-fabric/epoxy composites (FFRP) were also tested in the horizontal Underwriters Laboratories test: UL-94 HB. The rating (based on Table 3) results are presented in Table 5. The burning rates of the tested specimens are also presented in the table. Some specimens, however, immediately stopped burning after flame removal, or they extinguished between the 25 and 100 mm marks. Therefore, horizontal flame spreading rates could not be measured. The results showed that all tested specimens passed the UL-94 HB test. Composites containing APP and ALH fire retardants exhibited better fire properties in comparison to the untreated specimens. A rapid-fire spreading rate was also observed for the untreated specimen. Moreover, the flame-spreading rate could only be measured for the composite specimens with 10% APP and 20% ALH.

### 3.4. Tensile Tests

Tensile tests were carried out mainly to study the influence of the APP and ALH fire retardant compounds in the flax fibre-reinforced polymer (FFRP) composites. For each specimen type, at least five samples were successfully tested. The elastic modulus (E), strength (σ_u_), and maximum strain (ε_u_) as the results of the tensile tests are presented in Table 6. The elastic modulus was measured based on the linear stress–strain curved within the range of 10–35% of the strength. Based on the data, the results of the two-layer FFRP composites (2L-FFRP) were used as the reference.

The data showed that the elastic modulus, the strength, and the maximum strain of the one and the two layers of FFRP composites were relatively similar without any significant difference. A maximum difference of 4% was measured for the three mentioned parameters. When the fire-retardant compounds were present in the composite matrix, however, a reduction of the mechanical properties of the composites was observed. The average elastic modulus of the composites with 10% and 20% APP compounds reduced by 24% and 16%, respectively, in comparison to the composites without fire retardant compounds. Statistical analysis via t-test also showed a significant difference compared to the untreated specimen. Similar reductions were also observed for their strength values. Their maximum strains before failure slightly increased in comparison to the reference data. The composites containing 20%, 30%, and 40% ALH compounds also showed similar decreasing behaviours for their mechanical properties. The elastic moduli decreased by 16–21%, while their tensile strengths decreased by 3–14% compared to the specimen without fire retardants. On the contrary, their maximum strains increased on average by 60–90%, which was significantly different compared to the specimen without treatment. These results suggested that the addition of ALH fire-retardant compounds in the FFRP composite increased the ductility of the composites.

Based on these data, the optimum quantity of APP compounds was 20% for the tensile performance of FFRP composites. With this quantity, the elastic modulus and the strength of the composites were reduced by 16% and 18%, respectively, while insignificant changes could be observed for its maximum strain compared to the untreated composite. For the ALH compound, the optimum quantity was reached at 30% FR compound by mass content of the epoxy. The elastic modulus of this composite was reduced by 16%, with minimum reduction of strength. The maximum strain was increased by 90%, suggesting a more ductile material behavior, which is preferable for a material frequently used as a tension component. A ductile material will plasticize and show large visible deformations before failure, which is important for safety in building applications. In other studies (i.e., [26,50]), the addition of fire retardant to the fibre polymer matrix reduced the tensile strength of the composites by 16%, but the elastic modulus increased by 20%. With an additional treatment of the fibre yarn, such as alkali or saline treatment, and on the polymer matrix, such as nanoparticle treatment, the reduction of the elastic modulus and strength due to APP or ALH fire retardant treatments can be overcome [1].

### 3.5. SEM (Scanning Electron Microscope)

**Figure 5:**
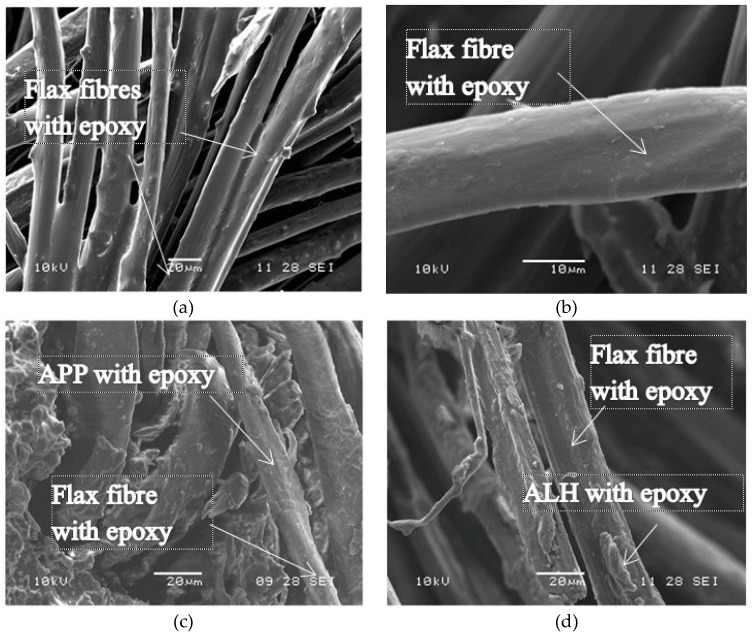
FFRP morphology using SEM: (**a,b**) 2L-FFRP (**c**) 2L-FFRP-APP30%; (**d**) 2L-FFRP-ALH40%.

The morphology characteristics of the composites were investigated using scanning electron microscopy. The goal was to obtain visible information related to fire resistance. The results are presented in Figure 5. Based on the images, flax fibres in the composites without fire retardant compounds showed smooth surfaces that were fully covered with the adhesive (Figure 5a,b). Flax fabric is a natural material and it is naturally hydrophilic. APP and ALH have been also reported as super hydrophilic materials with water contact angle measurement approaching 0° [53,54]. Thus, the polarity of these materials supports their compatibility in the composites. The fibres in the composite treated with flame retardants had an inhomogeneous surface structure (Figure 5c,d). This inhomogeneity was suspected because of the presence of flame retardants that were mixed with epoxy resin. The aggregates of the particulate fire retardants were attached onto the surface of the fibre, which reduced the contact area between the epoxy resin and the flax fibre. This was suspected to be the main reason for the reduction of the mechanical properties as discussed in the previous section.

## 4. Conclusions

In this work, the thermal stability and fire resistance of flax fabric-reinforced polymer composites were investigated. The thermal stability investigations revealed that the composite treatments using the two investigated fire retardants, APP and ALH, led to a substantial increase in their thermal resistance. Based on TGA analysis, APP and ALH had different impacts on the degradation rate of the material, which was related to their different fire prevention mechanisms. APP produces protective char to prevent further burning, while ALH produces water vapour to reduce the heat. In this test, the composites containing 30% APP and 30% ALH were the two composites with optimum thermal stability from the two fire retardants. Based on the LOI and the UL-94 V tests, better fire resistance was achieved by the composite treated with APP compounds. A higher APP content in the composite also led to a higher LOI value and UL-94V rating. The ALH composites, on the other hand, only passed the UL-HB rating. They performed poorly in the LOI test and did not pass the UL-94 V test. However, based on the mechanical test, the best performance was obtained by the composite with 30% ALH. Their average elastic modulus and strength were slightly reduced by 16% and 3%. Their maximum strains increased almost twofold in comparison to the untreated specimen, which suggested a more ductile behaviour preferable for the use of the composite as tension structural components. The SEM results showed the presence of fire-retardant compounds inside the composite matrix. It was also observed that the FR compound aggregated in the composite matrix. This was suspected as the main reason for the reduction in mechanical properties of the FR treated composite, as they reduced the fibre-epoxy contact area. The reduction of the mechanical properties can be overcome with additional treatment of the flax fibre and also with additional nanoparticles in the epoxy matrix. However, further study to ensure the compatibility of the materials is required.

## Figures and Tables

**Table 1:** Tested composites with corresponding abbreviations, thicknesses, and matrix content.

Specimen ID	Specimen name	Thickness [mm]	Fire Retardant (FR) content [%]
1L-FFRP	1 layer flax-fabric/epoxy laminate	1.5	
2L-FFRP	2 layers flax-fabric/epoxy laminate	3	-
4L-FFRP	4 layers flax-fabric/epoxy laminate	6	-
2L-FFRP-APP10%	2 layers flax-fabric/epoxy laminate, 10% APP	3	10% APP
2L-FFRP-APP20%	2 layers flax-fabric/epoxy laminate, 20% APP	3	20% APP
2L-FFRP-APP30%	2 layers flax-fabric/epoxy laminate, 30% APP	3	30% APP
2L-FFRP-ALH20%	2 layers flax-fabric/epoxy laminate, 20% ALH	3	20% ALH
2L-FFRP-ALH30%	2 layers flax-fabric/epoxy laminate, 30% ALH	3	30% ALH
2L-FFRP-ALH40%	2 layers flax-fabric/epoxy laminate, 40% ALH	3	40% ALH

FFRP: Flax flax reinforced polymer composite; APP: ammonium polyphosphate; ALH: aluminum hydroxide.

**Table 2:** Burning criteria for UL-94 vertical rating.

Test criteria	UL 94 V rating		
	V-0	V-1	V-2
Burning time of each individual test specimen (s) (after first and second flame applications)	≤10	≤30	≤30
Burning and afterglow times after second flame application (s)	≤30	≤60	≤60
Dripping of burning specimens (ignition of cotton batting)	No	No	Yes
Combustion up to holding clamp (specimens completely burned)	No	No	No

**Table 3:** Burning criteria for UL-94 horizontal rating.

Test criteria	Burning rate in V	UL 94 HB rating
Test specimen thickness 3–13 mm	≤40 mm/min	HB
Test specimen thickness <3 mm	≤75 mm/min	HB
Flame is extinguished before first mark	= 0 mm/min	HB

**Table 4:** Limited oxygen index (LOI) values of FFRP and other materials as the reference.

Materials	n ^a^	LOI ^b,c^ (%)		Literature
Pine wood (*Pinus sylvestris* L.)	3	22.0 (±4.5%)	NS	Own measurements
Beech wood (*Fagus sylvatica* L.)	3	22.7 (±6.7%)	NS	
2L-FFRP	3	21.3 (±2.7%)	**REF**	
4L-FFRP	4	23.3 (±7.3%)	NS	
2L-FFRP-APP10%	5	22.4 (±5.1%)	NS	
2L-FFRP-APP20%	6	25.5 (±12.1%)	S	
2L-FFRP-APP30%	3	30.3 (±6.9%)	S	
2L-FFRP-ALH20%	4	22.5 (±5.7%)	NS	
2L-FFRP-ALH30%	2	23.5 (±3.0%)	S	
2L-FFRP-ALH40%	2	24.5 (±2.9%)	S	
Hemp fabric/epoxy	-	22 (−)		[48]
Glass fabric/epoxy	-	21 (−)		[48]
Cabron fabric/epoxy	-	25 (−)		[49]
Glass fabric/polybutylene terephthalate		22 (−)		[50]
Glass fabric/polybutylene terephthalate-20% aluminum hypophosphite		29 (−)		[50]

^a^ Number of successfully tested specimens; ^b^ Coefficient of variance (%) in parentheses; ^c^ Statistical analysis for data comparison via t-test with a significance level of 0.05 (NS = no significant difference, S = significant difference compared to the untreated 2L-FFRP).

**Table 5:** Results of vertical (UL-94 V) and horizontal (UL-94 HB) burning tests.

Specimen ID	UL-94 V ^a^		UL-94 HB ^b^		
	n ^a^	Rating	n ^a^	Burning rate (mm/min)	Rating
2L-FFRP	5	NR	3	14.9 (4.1%)	HB
4L-FFRP	5	NR	3	8.3 (1.3%)	HB
2L-FFRP-APP10%	5	NR	3	18.6 (11.3%)	HB
2L-FFRP-APP20%	5	V-1	3		HB
2L-FFRP-APP30%	5	V-0	3		HB
2L-FFRP-ALH20%	5	NR	3	11.6 (11.5%)	HB
2L-FFRP-ALH30%	5	NR	3		HB
2L-FFRP-ALH40%	5	NR	3		HB

^a^ Number of successfully tested specimens; ^b^ Coefficient of variance (%) in parentheses.

**Table 6:** The results of the mechanical test under tension loading.

Specimen ID	n	E [MPa] ^a^	ΔE [%] ^b,c^		σ_u_ [MPa] ^a^	Δσ_u_ [%] ^b,c^		ε_u_ [%] ^a^	Δε_u_ [%] ^b,c^	
1L-FFRP	9	5180 (6.1%)	−4	NS	46 (6.5%)	−3	NS	1.4 (14.8%)	+3	NS
2L-FFRP	6	5420 (4.1%)	**REF**	**REF**	47 (5.1%)	**REF**	**REF**	1.3 (6.0%)	**REF**	**REF**
2L-FFRP-APP10%	5	4130 (9.1%)	−24	S	40 (3.9%)	−17	S	1.5 (19.6%)	+16	NS
2L-FFRP-APP20%	5	4530 (3.3%)	−16	S	39 (3.4%)	−18	S	1.3 (12.1%)	−1	NS
2L-FFRP-ALH20%	5	4290 (4.3%)	−21	S	42 (4.1%)	−11	S	2.1 (12.5%)	+60	S
2L-FFRP-ALH30%	5	4570 (2.6%)	−16	S	46 (3.9%)	−3	NS	2.5 (5.8%)	+90	S
2L-FFRP-ALH40%	5	4460 (3.6%)	−18	S	41 (4.1%)	−14	S	2.5 (10.4%)	+86	S

^a^ Coefficient of variance (%) in parentheses; ^b^ Delta (Δ) indicates the difference of the average value to the reference 2L-FFRP (plus (+) and minus (−) indicate if the value is higher or lower in respect to the reference, respectively); ^c^ Statistical analysis for data comparison via t-test with a significance level of 0.05 (NS = no significant difference, S = significant difference compared to the untreated 2L-FFRP).

## Nomenclature

ALH	-	Aluminium hydroxide, Al(OH)_3_
APP	-	Ammonium polyphosphate, NH_4_PO_3_
CO	-	Carbon monoxide
FR	-	Fire retardant
FFRP	-	Flax fabric reinforced polymer composite
HCl	-	Hydrogen chloride
HCN	-	Hydrogen Cyanide
HF	-	Hydrogen Fluoride
L	-	Layer
LOI	-	Limited oxygen index
NFRP	-	Natural fibre reinforced polymer
NS	-	No significant difference to the reference data
SEM	-	Scanning electron microscope
SPR	-	Smoke production rates
S	-	Significant difference to the reference data
TGA	-	Thermogravimetric analysis
TTI	-	Time to ignition
UL	-	Underwriters Laboratories
